# GABAB1R: A molecular bridge linking deep brain stimulation to antidepressant effects——A commentary on “NAcLat-DBS reduces GABAB1R expression of dopamine neurons to correct depressive symptoms in CMS mice"

**DOI:** 10.1016/j.neurot.2026.e01055

**Published:** 2026-08-29

**Authors:** Fang Li, Jing Sun, Ming-Hu Han

**Affiliations:** a The Brain Cognition and Brain Disease Institute, Shenzhen Institutes of Advanced Technology (SIAT), Chinese Academy of Sciences, Shenzhen, 518055, China; b Department of Mental Health and Public Health, Faculty of Life and Health Sciences, Shenzhen University of Advanced Technology (SUAT), Shenzhen, 518107, China; c Department of Pharmacological Sciences, Friedman Brain Institute, Icahn School of Medicine at Mount Sinai, New York, NY, 10029, USA

**Keywords:** Deep brain stimulation, GABAB1 receptor, Dopamine neurons, Ventral tegmental area, Nucleus accumbens

Treatment-resistant depression (TRD) remains one of the most formidable challenges in clinical psychiatry. Despite the wide array of antidepressant medications, approximately one-third of patients with major depressive disorder (MDD) do not achieve a satisfactory response to these agents [1]. In this context, the neuromodulatory intervention of deep brain stimulation (DBS) offers new hope for patients with MDD. Nucleus accumbens (NAc) and its subregions have been explored as targets for DBS and have shown promising antidepressant effects in clinical investigations [2].

In the field of movement disorders, the therapeutic mechanism of DBS is generally attributed to acute modulation of local pathological oscillatory activity by high-frequency electrical stimulation [3,4]. However, treatment of depression rarely exhibits such an immediate on/off effect, and clinical studies have shown that DBS typically requires weeks or even months of continuous stimulation to achieve stable mood improvement, with therapeutic effects persisting for prolonged periods of time even after cessation of stimulation [5,6]. This unique kinetic profile was documented in early studies, which reported that progressively longer periods of continuous stimulation result in a correspondingly longer carry-over of beneficial behavioral effects after cessation of stimulation [7]. This implies that the effects of DBS in psychiatric disorders involves enduring fundamental changes. Indeed, clinical evidence that NAc DBS can alleviate anhedonia and depressive symptoms in a subset of patients with treatment-resistant depression has accumulated for nearly two decades [8,9]. However, how the stimulation reshapes the functional state of neurons to produce sustained mood improvement remains without a systematic molecular explanation to date—precisely the gap that Jing et al. aim to fill in this issue of *Neurotherapeutics* [10].

Jing and colleagues employed a mouse model of chronic mild stress (CMS) and found that long-term DBS targeting the lateral shell of the nucleus accumbens (NAcLat) significantly downregulated the expression of inhibitory GABAB1 receptors (GABAB1R) in ventral tegmental area (VTA) dopamine neurons, thereby restoring dopaminergic activity and reversing depressive-like behaviors. This discovery shifts the mechanistic understanding of DBS from “acute circuit-level modulation” to “long-term receptor-molecular remodeling,” providing critical new insight into the therapeutic efficacy of DBS.

Notably, two weeks of sustained NAcLat DBS did not merely transiently alter the excitability of VTA dopamine neurons, but rather induced structural remodeling of G protein-coupled receptors at the level of membrane expression. Downregulation of GABAB1R was not a transient functional inhibition, but rather was a sustained reduction in the surface availability of the receptor protein. Shifting the therapeutic effects of DBS from “functional modulation” to the level of “synaptic receptor remodeling” suggests that DBS may reset the disrupted excitation–inhibition balance in depression through mechanisms akin to long-term synaptic plasticity or homeostatic scaling. The experimental design of Jing et al. was particularly rigorous. They not only observed a robust correlation between decreased GABAB1R expression following DBS and behavioral improvement, but also constructed a complete causal chain through four orthogonal approaches of agonist activation, antagonist blockade, targeted overexpression, and specific knockdown. Activation of GABAB receptors with the pan-GABAB receptor agonist baclofen induced canonical depressive-like behaviors in naive mice; blockade with the pan-GABAB receptor antagonist CGP35348 recapitulated the antidepressant effects of DBS in CMS mice; and specific overexpression of GABAB1R in VTA dopamine neurons completely reversed the behavioral benefits of DBS, with targeted knockdown of the receptor conferring protective effects. These four converging lines of evidence establish a central role of GABAB1R in the antidepressant mechanism of DBS. Crucially, the bidirectional genetic manipulations extend the significance of GABAB1R beyond that of a mere therapeutic effector. The observation that specific knockdown of this receptor confers resilience to chronic stress, while its overexpression accelerates depressive phenotypes, aligns with the modern neurobiological framework of stress vulnerability, establishing GABAB1R expression in dopamine neurons as a critical molecular switch governing susceptibility versus resilience to environmental adversities [11].

While we affirm the principal findings of this study, one experimental result merits particular attention. Although DBS significantly reduced GABAB1R expression in dopamine neurons, Gabbr1 mRNA levels in VTA GABA neurons showed no significant change. This finding rules out the possibility that DBS-mediated downregulation of GABAB1R represents a global, non-specific effect across the entire VTA, at least for GABAergic neurons. Furthermore, the authors observed dephosphorylation of the GABAB2 subunit at Ser-783. Although these two sets of experiments examined different dimensions—the former assessing mRNA levels and the latter assessing membrane protein phosphorylation status—the two findings are mechanistically complementary, with unchanged mRNA levels alongside decreased membrane protein expression suggesting that GABAB1R downregulation occurs primarily at the post-translational level, while dephosphorylation at Ser-783 of the GABAB2 subunit provides a potential molecular clue for this post-translational regulation. These two observations reinforce each other.

If the downregulation of GABAB1R represents the “final common pathway” of the antidepressant effects of DBS, a further upstream question arises of what signal mediates this DBS-induced downregulation? The authors provide a particularly illuminating answer here: glutamate. Supporting this conclusion, direct *in vivo* recordings with an iGluSnFR glutamate sensor revealed that NAcLat DBS induced a significant increase in extracellular glutamate release in the VTA, alongside a marked reduction in phosphorylation levels of the GABAB2 subunit at Ser-783. Notably, glutamate-driven GABAB receptor endocytosis has been documented previously [12], and dephosphorylation at Ser-783 suggests a potential mechanism for regulating receptor membrane stability. This observation integrates the glutamate, GABA, and dopamine systems, implying that dopaminergic hypofunction in depression may not represent an isolated monoamine deficit, but rather a secondary consequence of glutamate-GABA imbalance. Importantly, this interpretation complements the previous finding that “NAc-DBS disinhibits dopamine neurons by suppressing local GABAergic interneurons in the VTA” [13]: GABAergic disinhibition provides an “acute” driving force for increased dopamine neuron excitability, whereas enhanced glutamatergic input may further trigger the “chronic” downregulation of GABAB1R membrane expression. The temporal dissociation between these two mechanisms precisely mirrors the time lag between “immediate neurophysiological changes” and “gradual behavioral improvement,” which is characteristic of DBS antidepressant effects.

Although the findings of Jing et al. were derived from mouse models, their potential for clinical translation points to at least three directions. First, the observation that the pan-GABAB receptor antagonist CGP35348 can pharmacologically recapitulate the primary behavioral benefits of DBS, despite showing only a non-significant trend in rescuing global dopamine release when administered systemically, suggests that generalized GABAB antagonism may offer a pharmacological alternative for surgically ineligible patients. However, the widespread distribution of these receptors would require that future compounds have sufficient brain-region specificity to minimize systemic side effects. Indeed, the clinically tested GABAB receptor antagonist SGS742 has provided preliminary evidence of good tolerability in human clinical trials investigating cognitive impairment [14]. Although this trial focused on cognitive impairment rather than depression, it provides an important safety precedent for pursuing this therapeutic direction. Second, the efficacy of DBS may depend on whether baseline GABAB1R is in a state amenable to downregulation, providing a potential biomarker strategy for identifying optimal responders, although no mature GABAB1R-targeted PET tracer is currently available for routine clinical use. Third, to address the clinical challenge of delayed therapeutic onset of DBS, a combined strategy of DBS with a pan-GABAB receptor antagonist may accelerate therapeutic onset or enhance efficacy through the synergistic interaction between active endocytosis and functional blockade. Clinical validation of these hypotheses will require systematic investigation, and these findings have charted clear directions for subsequent translational efforts.

There are some limitations of this study. First, they used only male CMS mice. Given that depression has a higher prevalence in females and GABA receptor function is modulated by estrogen, the generalizability of these conclusions awaits validation in female animals. Second, as acknowledged by the authors, glutamate-driven GABAB1R endocytosis currently serves as a correlational “working hypothesis.” The lack of specific endocytosis markers (e.g., EEA1) means that the establishment of its strict causality still requires direct validation through targeted NMDA/AMPA receptor blockade experiments during DBS. Third, while viral manipulations confirm dopamine neuron specificity, the bulk VTA membrane protein assays cannot completely exclude the potential contribution of astrocytic GABAB receptor shifts, which reflect a glial-neuronal interplay known to heavily influence synaptic transmission [15]. Furthermore, the post-endocytic fate of these receptors, the differential effects of varying DBS parameters, and the replicability of these findings in human TRD patients all remain unaddressed.

These limitations do not diminish the value of the current study. Rather, they identify the most promising directions for future inquiry. Jing and colleagues have revealed, through rigorous experimental evidence, a novel mechanistic layer underlying the antidepressant effects of NAcLat-DBS: enhanced glutamatergic input in the VTA downregulates membrane expression of inhibitory GABAB1 receptors (GABAB1R) on dopamine neurons, thereby restoring mesolimbic dopaminergic activity and reversing both behavioral despair and anhedonia in CMS mice (Fig. 1). The core scientific contribution of this work lies in advancing our understanding of DBS therapeutic effects from the physical level of “currents and firing” to the molecular biological level of “receptors and synapses"—the sustained mood improvement produced by DBS may depend not merely on its acute modulation of neural circuit excitability, but more fundamentally on its capacity to drive long-term remodeling of synaptic receptor networks. From the perspective of clinical translation, this study points to a novel pharmacological target for TRD treatment, provides a potential biomarker framework for personalized DBS application, and opens avenues for exploring combined strategies of neuromodulation and pharmacotherapy. While the journey from CMS mice to TRD patients will be undeniably long, the establishment of GABAB1R as a molecular hub has already set a clear and promising landmark for the road ahead.

**Fig. 1:**
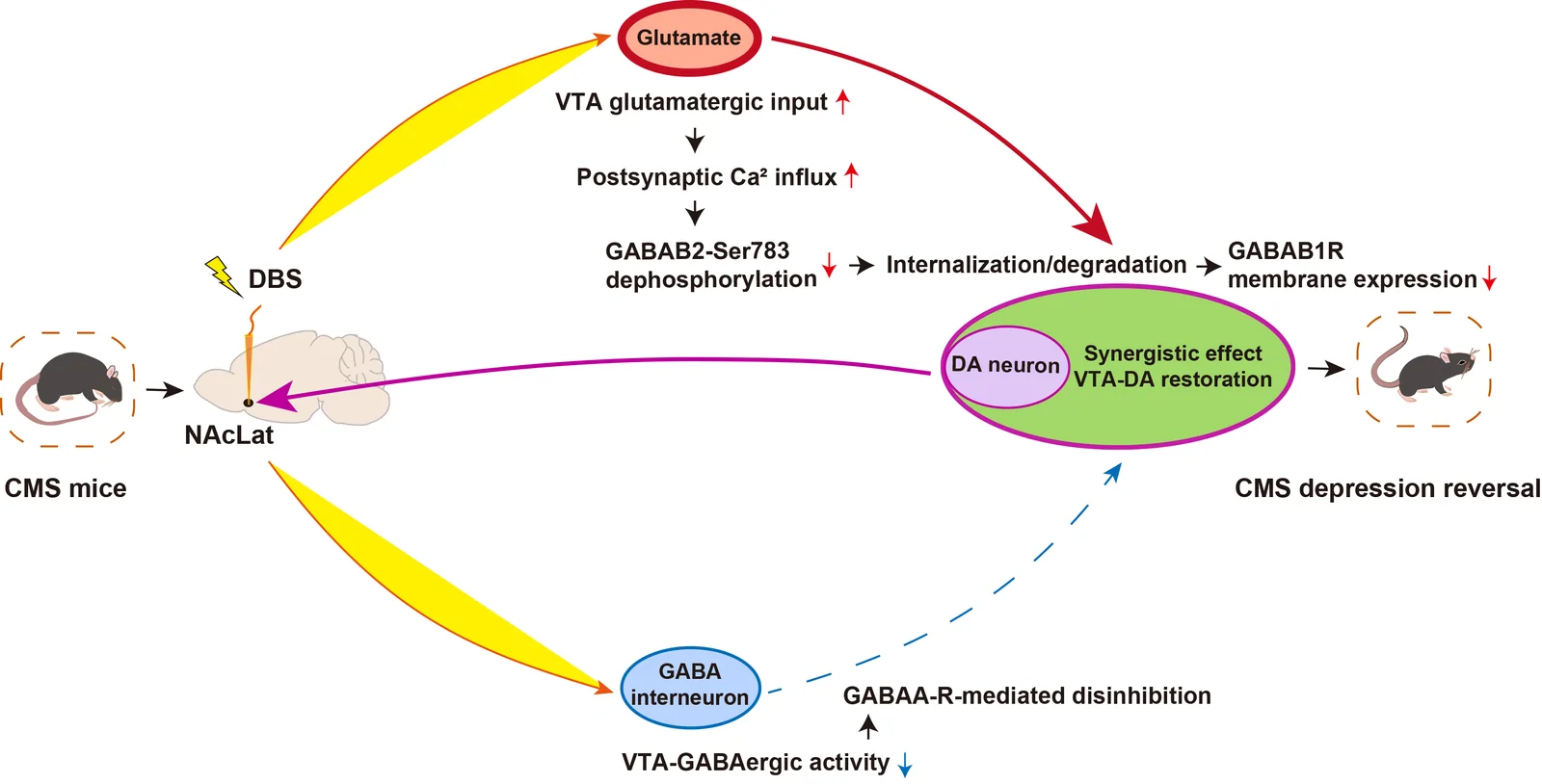
NAcLat-DBS reverses depressive-like behaviors in CMS mice through a dual-layer mechanism of “acute disinhibition and chronic receptor remodeling.”

Jing et al. demonstrated that NAcLat-DBS reduces GABAB1R expression in VTA dopamine (DA) neurons, and this downregulation is required for its antidepressant effects. Mechanistically, this reduction is achieved through two parallel pathways operating on distinct timescales: one involves suppression of local VTA GABAergic activity, leading to rapid disinhibition of dopamine neurons [13]; the other involves enhanced VTA glutamatergic input, accompanied by GABAB2-Ser783 dephosphorylation, driving sustained downregulation of GABAB1R membrane expression. Pharmacological blockade of GABAB receptors by the pan-GABAB antagonist CGP35348 reproduced the antidepressant-like effects of DBS, whereas selective overexpression of GABAB1R in the VTA–NAcLat pathway reversed the behavioral benefits. Collectively, this study establishes GABAB1R as a critical molecular hub linking DBS to dopamine restoration and suggests that modulation of VTA glutamatergic excitation represents a potential pharmacological strategy for treatment-resistant depression.

## Author contributions

Fang Li and Jing Sun contributed equally to this work as co-first authors. Both authors jointly conceptualized the Commentary, surveyed and synthesized the relevant literature, drafted the manuscript, and participated in its revision. Ming-Hu Han supervised the project, provided critical intellectual guidance, and revised the manuscript. All authors read and approved the final version for submission.

## Funding

This work was supported by the National Key R&D Program of China (Grant Nos. 2021ZD0202900 and 2021ZD0202902), Shenzhen Medical Research Fund (Grant No. B2302012), Guangdong Basic and Applied Basic Research Foundation (Grant No. 2026A1515012803).

## Declaration of competing interest

The authors declare that they have no known competing financial interests or personal relationships that could have appeared to influence the work reported in this paper.
